# Association between anxiety and aggression among students at a medical college in China: a cross-sectional analysis of indirect associations involving physical activity motivation and self-regulatory fatigue

**DOI:** 10.3389/fpsyg.2026.1916035

**Published:** 2026-08-20

**Authors:** Tianyu Zhao, Rui Dong, Qiqi Yin

**Affiliations:** 1 Faculty of Nursing, Shangqiu Medical College, Shangqiu, Henan, China; 2 Department of Physical Education, Shangqiu Medical College, Shangqiu, Henan, China

**Keywords:** aggression, anxiety, China, cross-sectional study, medical college students, physical activity motivation, self-regulatory fatigue, statistical indirect association

## Abstract

**Background:**

Anxiety and aggression are important concerns for college students’ mental health and campus adjustment. However, motivational and self-regulatory correlates of this association remain insufficiently examined.

**Objective:**

This study examined the anxiety–aggression association among students at one medical college in China, tested separate and serial statistical indirect associations involving physical activity motivation (PAM) and self-regulatory fatigue (SRF), and examined the reverse ordering of PAM and SRF.

**Methods:**

A cross-sectional questionnaire survey was conducted among 1,773 students at Shangqiu Medical College, Henan Province, China. Measures included a GAD-7-derived anxiety item set with five-point agreement responses and no two-week recall period, a BAQ-derived aggression item set with five rather than seven response categories, and PAM and SRF scales. Pearson correlations, regression analyses, and PROCESS Model 6 with 5,000 percentile bootstrap samples were used. Gender and three age-group indicator variables were included as covariates; reverse-order and outlier sensitivity analyses were also conducted.

**Results:**

Anxiety showed modest correlations with aggression (*r* = 0.260), SRF (*r* = 0.254), and PAM (*r* = −0.257; all *p* < 0.001). The adjusted aggression model explained 14.5% of the variance (R^2^ = 0.145), and the direct anxiety–aggression association remained statistically distinguishable from zero (*B* = 0.1101, 95% CI [0.0807, 0.1395]). Bootstrap confidence intervals excluded zero for the separate statistical indirect associations involving PAM and SRF and for the prespecified serial association. The serial association was small (*B* = 0.0053, 95% bootstrap CI [0.0034, 0.0077]; completely standardized estimate = 0.0083, 95% bootstrap CI [0.0053, 0.0120]), and the reverse ordering yielded a similarly small serial association. The outlier sensitivity analysis did not materially alter the findings.

**Conclusion:**

Among students at one medical college, anxiety, lower PAM, higher SRF, and aggression formed modest cross-sectional covariance patterns. Because both PAM–SRF orderings were compatible with the data and the serial association was small, the findings are hypothesis-generating and do not establish temporal or causal mechanisms.

## 1 Introduction

The college years mark an important transition from structured educational environments to broader adult social roles. During this period, academic demands, interpersonal evaluation, peer comparison, and uncertainty about the future may expose students to persistent psychological stress. Anxiety and aggression are both important concerns for college students’ mental health and campus adjustment. Anxiety involves persistent worry, threat anticipation, and emotional tension, whereas aggression-related tendencies include physical and verbal aggression, anger, and hostility (Anderson and Bushman, 2002; Spitzer et al., 2006). Research among Chinese college students has reported an association between social anxiety and online aggressive behavior, indicating that the anxiety–aggression association is relevant in higher-education settings (Ding and Xu, 2025). However, the psychological variables involved in the anxiety–aggression association remain insufficiently examined, particularly in Chinese higher-education settings. Few studies have considered physical activity motivation (PAM) and self-regulatory fatigue (SRF) together as complementary motivational and self-regulatory correlates of this association.

To address this gap, PAM and SRF offer two complementary, theory-informed perspectives on the anxiety–aggression association. PAM refers to students’ reasons for engaging in or maintaining physical activity and reflects goal orientation, enjoyment, perceived competence, and social connectedness; it is distinct from actual physical activity level. Research on physical activity guided by Self-Determination Theory emphasizes the importance of motivational quality and psychological need satisfaction in initiating and maintaining physical activity (Ntoumanis and Moller, 2025). From this perspective, higher anxiety may covary with lower PAM and less favorable motivation-related experiences in physical-activity contexts. SRF refers to perceived fatigue in sustaining cognitive, emotional, and behavioral regulation. Related research has suggested that perceived stress is associated with SRF, indicating that SRF may be an important variable for understanding stress, negative emotions, and behavioral adjustment (Chang et al., 2026). A theory-compatible pattern is that higher anxiety covaries with higher SRF and higher SRF covaries with greater aggression. For the serial model, PAM was placed before SRF as one theory-compatible, prespecified ordering because lower PAM may covary with greater regulatory fatigue; however, the reverse ordering is also plausible because greater fatigue may reduce willingness to initiate or maintain physical activity. Given the cross-sectional questionnaire data, this ordering and the resulting statistical indirect associations describe covariance patterns only and do not establish temporal precedence or causal mechanisms. Accordingly, among students at one medical college in China, the present study aimed to: (1) examine the anxiety–aggression association; (2) estimate single-intermediary statistical indirect associations involving PAM and SRF; and (3) estimate the prespecified serial association involving PAM followed by SRF and examine the reverse ordering as a robustness analysis. The following section develops H1–H4 and the conceptual rationale for these analyses.

## 2 Literature review and research hypotheses

### 2.1 Anxiety and aggression

Anxiety refers to persistent worry, nervousness, threat anticipation, and uncertainty, whereas aggression encompasses physical, verbal, affective, cognitive, and interpersonal tendencies related to harming or confronting others (Anderson and Bushman, 2002; Spitzer et al., 2006). Aggression may therefore be reflected in physical aggression, verbal aggression, anger, hostility, or relational forms. According to the General Aggression Model, personal and situational factors are proposed to shape aggressive responses through internal states such as affect, cognition, and arousal. Within this framework, heightened arousal, threat sensitivity, and hostile interpretation provide a theory-compatible explanation for why anxiety may covary with irritability, defensive responding, and aggressive tendencies under interpersonal or evaluative stress (Anderson and Bushman, 2002).

Empirical research has also provided evidence for the association between anxiety and aggression. Previous studies have shown that higher anxiety is associated with higher levels of aggression and that anxiety is closely related to aggression-related dimensions such as anger and hostility. These findings suggest that anxiety-related experiences may not only be expressed as avoidance or withdrawal but may also be accompanied by anger, hostility, and aggressive responses (Chung et al., 2019; Florek et al., 2021). In contexts involving social evaluation and intimate relationships, social anxiety may also be associated with dating aggression, suggesting that individuals with higher anxiety may be more likely to show defensive or conflict-related responses when they perceive negative evaluation or relational threat (Hanby et al., 2012). In addition, aggression among students may appear in more covert interpersonal forms. Previous research has found that anxiety is associated with relational aggression, with both self-reported and peer-reported relational aggression reflecting this tendency; the association between social anxiety and relational aggression has also received attention in college student samples (Zimmer-Gembeck and Pronk, 2012; Deason et al., 2019). Anxiety symptoms may also be involved in the reciprocal relationship between aggression and peer victimization, suggesting that anxiety may be embedded in cycles of interpersonal conflict and peer interaction among students (Cooley et al., 2017). In Chinese higher-education settings, academic competition, interpersonal evaluation, peer comparison, and future uncertainty may be relevant contextual correlates, although these processes were not measured in the present study. Accordingly, this study proposed the following hypothesis:

Hypothesis 1 (*H1*): Anxiety is positively associated with aggression.

### 2.2 Statistical indirect association involving physical activity motivation

Physical activity motivation (PAM) is one of the two prespecified statistical intermediary variables considered in this study. It refers to individuals’ reasons for engaging in, maintaining, and investing effort in physical activity, including enjoyment, value endorsement, health goals, perceived competence, social connectedness, and willingness to continue participation. Importantly, PAM is not equivalent to actual physical activity level; it concerns why individuals are willing to participate in or maintain physical activity rather than how much physical activity they have completed. According to Self-Determination Theory, the satisfaction of autonomy, competence, and relatedness is an important psychological basis for high-quality motivation. When individuals experience autonomous choice, perceived competence, and social connectedness during an activity, they are more likely to develop autonomous motivation and sustain active participation (Ryan and Deci, 2000). In the field of physical activity, research based on Self-Determination Theory has also shown that autonomous motivation, perceived competence, and intrinsic interest are closely associated with participation and persistence in exercise and physical activity (Teixeira et al., 2012). Anxiety-related threat anticipation, competence concerns, and sensitivity to physical or social evaluation may be associated with less favorable autonomy, competence, and relatedness experiences in physical-activity contexts, providing a theory-compatible basis for expecting higher anxiety to covary with lower PAM. Related studies have also shown that autonomy support can promote more self-determined behavioral regulations and physical activity intention, whereas anxiety-related experiences such as social physique anxiety may weaken individuals’ autonomous and competence-related experiences in physical activity (Lim and Wang, 2009; Brunet and Sabiston, 2009).

Furthermore, PAM may not only reflect students’ willingness to engage in physical activity but may also be understood as a positive motivational resource. Previous research has shown that physical activity motivation is not a unitary construct; rather, it may be represented by different profiles, such as low motivation, controlled motivation, and autonomous motivation. These motivational profiles differ in physical activity level, intention to continue participation, and subjective experience (Friederichs et al., 2015). Among college students, physical activity motive profiles are also closely associated with psychological need satisfaction, indicating that physical activity motivation is strongly linked to positive psychological experiences such as autonomy, competence, and relatedness (Valenzuela et al., 2021). From this perspective, higher PAM may be associated with more favorable physical-activity-related goals, enjoyment, perceived competence, and social connectedness, although PAM does not establish actual activity participation or psychological resource gain. Accordingly, lower PAM may form part of the covariance pattern linking anxiety and aggression, without implying that anxiety temporally reduces PAM or that PAM causes aggression. Accordingly, this study proposed the following hypothesis:

Hypothesis 2 (*H2*): Higher anxiety is associated with lower physical activity motivation, and lower physical activity motivation is associated with higher aggression; accordingly, the anxiety–aggression association includes a statistical indirect association involving physical activity motivation.

### 2.3 Statistical indirect association involving self-regulatory fatigue

Self-regulatory fatigue (SRF) is the second prespecified statistical intermediary variable considered in this study. It refers to regulatory fatigue experienced during sustained cognitive, emotional, and behavioral regulation and is typically reflected in reduced regulatory capacity at cognitive, emotional, and behavioral levels. Previous scale development research has shown that SRF can be distinguished from physical fatigue and general self-control, indicating that it is not merely physical fatigue and cannot simply be equated with self-control in a general sense (Nes et al., 2013). Theoretically, SRF is closely related to ego depletion and self-control failure, but it is not limited to a short-term depletion response following laboratory self-control tasks. Rather, it represents a regulatory fatigue state involving the joint operation of physiological, psychological, and motivational factors (Evans et al., 2016). According to the Strength Model of Self-Control, individuals expend limited self-regulatory resources when engaging in emotional control, attentional regulation, impulse inhibition, and response adjustment, and sustained effortful regulation may increase subsequent regulatory difficulties (Baumeister et al., 2007). Although this model has been debated and revised in later research, its account of self-regulatory resource load still provides an important theoretical basis for understanding the association between anxiety and SRF (Baumeister et al., 2018). Persistent worry, threat anticipation, emotional arousal, and attentional demands provide a theory-compatible basis for expecting higher anxiety to covary with higher SRF.

Furthermore, the association between SRF and aggression can be understood from the inhibition component proposed in I^3^ Theory. I^3^ Theory suggests that whether aggression is expressed depends not only on instigation and impellance, but also on whether individuals have sufficient inhibition; when inhibitory capacity is weaker, aggressive impulses may be more likely to be expressed as aggressive responses (Finkel, 2014). From this perspective, higher SRF may covary with weaker inhibition, impulse control, or response delay under frustration or conflict; these processes provide a theoretical interpretation rather than variables measured directly in the present study. Related reviews have also shown a close association between self-control failure and aggression, suggesting that lower self-control or regulatory failure is often associated with higher aggression, whereas stronger self-control has been discussed in relation to lower aggressive responses (Denson et al., 2012). However, existing research has more often explained aggression from the perspectives of general self-control, ego depletion, or inhibition failure, and has less frequently incorporated SRF as an independent statistical mediator in models of the association between anxiety and aggression. Accordingly, this study proposed the following hypothesis:

Hypothesis 3 (*H3*): Higher anxiety is associated with higher self-regulatory fatigue, and higher self-regulatory fatigue is associated with higher aggression; accordingly, the anxiety–aggression association includes a statistical indirect association involving self-regulatory fatigue.

### 2.4 Prespecified serial statistical indirect association involving physical activity motivation and self-regulatory fatigue

Beyond the two single-intermediary associations proposed above, PAM and SRF were also specified in a serial statistical ordering within the anxiety–aggression model. From a Self-Determination Theory perspective, anxiety-related threat, competence concerns, and social-evaluative sensitivity may covary with less favorable physical-activity motivation. Related research has shown that anxiety in sport discontinuation or return-to-sport contexts is associated with sport motivation, and that mental health problems may function as both a motivator for and a barrier to physical activity, indicating that anxiety-related experiences may have a complex association with physical activity motivation (Ruffault et al., 2020; Marashi et al., 2021). Conservation of Resources Theory further proposes that stress and regulatory difficulty are more likely when valued resources are threatened or difficult to replenish (Hobfoll, 1989). In the present framework, PAM is treated as a motivational correlate of valued physical-activity experiences, whereas SRF reflects perceived difficulty sustaining cognitive, emotional, and behavioral regulation; thus, lower PAM may covary with higher SRF. The reverse ordering is also theoretically plausible because greater SRF may reduce willingness to initiate or maintain physical activity. Research in sport psychology and self-control has also suggested that physical activity persistence and sport performance often involve self-control strength, and that theoretical links exist among motivational quality, autonomy support, vitality, and depletion. These findings provide further support for the serial association between PAM and SRF (Englert, 2016; Muraven, 2008; Muraven et al., 2008).

For the latter part of the model, I^3^ Theory provides a rationale for an association between higher SRF and greater aggression through reduced inhibition under instigation or impellance (Finkel and Hall, 2018). Accordingly, the prespecified model represents a statistical sequence in which higher anxiety covaries with lower PAM, lower PAM covaries with higher SRF, and higher SRF covaries with greater aggression. PAM was placed before SRF as one theory-compatible, prespecified ordering, not because temporal precedence had been established; the reverse ordering remained plausible and was examined as a competing model. Accordingly, this study proposed the following hypothesis:

Hypothesis 4 (*H4*): The anxiety–aggression association includes a serial statistical indirect association involving lower physical activity motivation followed by higher self-regulatory fatigue within the prespecified model.

**Figure 1:**
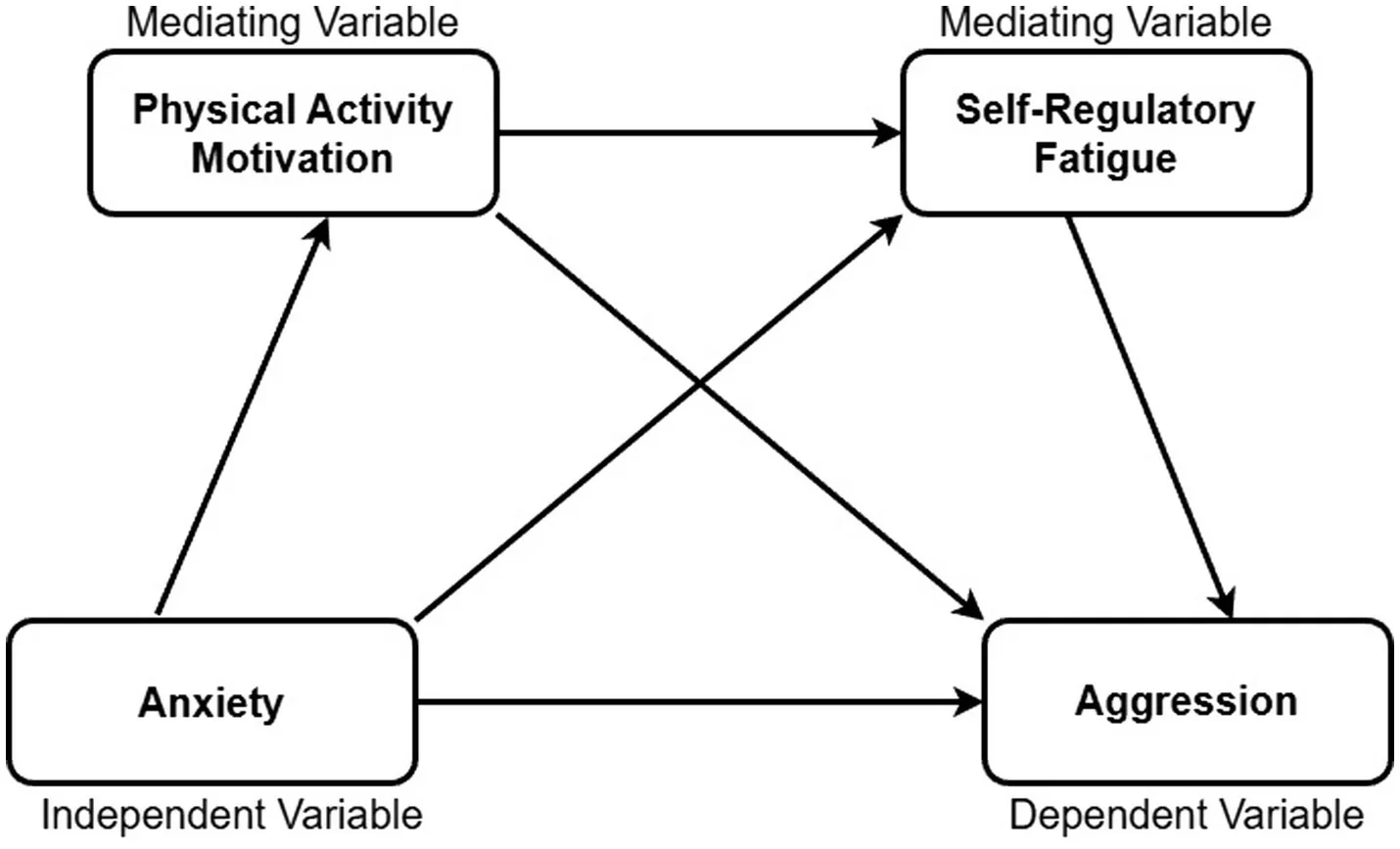
Conceptual framework of the prespecified cross-sectional statistical association model. The model specifies a positive association between anxiety and aggression, two single-intermediary statistical indirect associations involving physical activity motivation and self-regulatory fatigue, and one prespecified serial statistical indirect association involving physical activity motivation followed by self-regulatory fatigue. The arrows denote hypothesized statistical associations within the cross-sectional model and do not imply causal effects or temporal precedence.

Taken together, the conceptual framework specifies a positive anxiety–aggression association, two single-intermediary statistical indirect associations, and one prespecified serial statistical indirect association. Figure 1 summarizes the four hypotheses and the prespecified statistical ordering involving anxiety, PAM, SRF, and aggression. The framework is intended to organize cross-sectional covariance patterns and does not establish temporal precedence or causal mechanisms; competing orderings remain possible.

## 3 Materials and methods

### 3.1 Participants

This cross-sectional questionnaire survey was conducted from May 16 to May 26, 2026, at Shangqiu Medical College, Henan Province, China. Convenience sampling combined with classroom-based group recruitment was used. Class advisors and course instructors assisted with distributing the Wenjuanxing questionnaire link, and students completed it independently and voluntarily during class breaks; teaching staff did not assist with questionnaire completion or access individual responses. A total of 1,800 students were provided access to the questionnaire. After 27 invalid submissions were excluded, 1,773 valid questionnaires were retained, yielding a valid-questionnaire rate of 98.50%. The sample included 855 male students (48.2%) and 918 female students (51.8%). In terms of age, 285 students were 18 years old (16.1%), 557 were 19 years old (31.4%), 543 were 20 years old (30.6%), and 388 were 21 years old or older (21.9%). Regarding grade level, 792 participants were first-year students (44.7%), 644 were second-year students (36.3%), and 337 were third-year students (19.0%). The inclusion criteria were as follows: (1) being a current student at Shangqiu Medical College during the survey period; (2) being 18 years of age or older; (3) being able to understand the questionnaire and complete it independently; and (4) voluntarily agreeing to participate in the study and providing informed consent. The exclusion criteria were as follows: (1) failure to provide informed consent; (2) not meeting the eligibility criteria for the study population; (3) incomplete questionnaire submission or missing responses that prevented calculation of one or more core scale scores; (4) suspected duplicate submission; (5) clearly implausible completion time identified during data cleaning; and (6) extended repetitive, patterned, or straight-line responding. These criteria were used to improve data quality. The excluded submissions were deleted during data cleaning, and the exact completion-time threshold and category-specific exclusion counts were not retained. This study was conducted in accordance with the ethical principles for research involving human participants set out in the Declaration of Helsinki and was approved by the Medical Research Ethics Committee of Shangqiu Medical College, with the approval number SYLL 2026-035. Before completing the questionnaire, all participants were informed of the study purpose, anonymity, confidentiality, voluntary participation, and their right to withdraw at any time. The online questionnaire could be completed only after electronic informed consent had been confirmed, and the same requirement applied when the survey was administered in classroom settings. All questionnaire data were used only for research purposes, and directly identifiable personal information was not collected or included in the statistical analyses.

### 3.2 Measures

Chinese-language wording was used for all questionnaire items. The present study did not conduct a formal forward–back translation or independent pilot-validation procedure for the adapted item sets. Scale scores were calculated as item means, with higher scores indicating higher levels of the corresponding constructs. CFA was used to evaluate a single-factor anxiety model and second-order models of physical activity motivation, self-regulatory fatigue, and aggression, with *χ*^2^/df, RMSEA, CFI, and TLI used as fit indices.

#### 3.2.1 Anxiety

Anxiety was measured using a seven-item GAD-7-derived item set based on the core content of the Generalized Anxiety Disorder 7-item Scale (Spitzer et al., 2006). Sample items include “Feeling nervous, anxious, or on edge” and “Not being able to stop or control worrying.” The original GAD-7 uses a 0–3 frequency response scale. The original two-week recall period was not retained in the administered questionnaire. To standardize the response format across the questionnaire, this study modified the response options to a 5-point agreement scale, ranging from 1 = “strongly disagree” to 5 = “strongly agree.” Therefore, the original GAD-7 cut-off scores were not used for anxiety screening or diagnostic interpretation in this study. Instead, anxiety was treated as a continuous self-report indicator for statistical analysis. The mean score of the seven items was calculated, with higher scores indicating higher levels of anxiety-related experiences. In the present sample, the item set showed good internal consistency, and the CFA results indicated good measurement model fit: Cronbach’s *α* = 0.892, *χ*^2^/df = 2.898, RMSEA = 0.033, CFI = 0.995, TLI = 0.993. These results support internal consistency and structural coherence in the present sample but do not establish equivalence with the original GAD-7 or criterion validity.

#### 3.2.2 Physical activity motivation

Physical activity motivation was measured using a shortened Chinese-language adaptation of the Motives for Physical Activity Measure–Revised (MPAM-R), which assesses individuals’ motives for engaging in physical activity (Ryan et al., 1997; Chen et al., 2013). The scale contains 15 items across five dimensions: Health/Fitness, Appearance, Enjoyment/Interest, Competence, and Social, with three items in each dimension. Sample items include “Because I want to be physically fit” and “Because it’s fun.” To standardize the response format across the questionnaire, this study used a 5-point Likert scale, ranging from 1 = “strongly disagree” to 5 = “strongly agree.” The resulting scores were used only as continuous indicators of physical activity motivation in the present sample and were not directly compared with scores from the original MPAM-R. Because this study focused on overall physical activity motivation, the mean score of the 15 items was used as the composite score in subsequent statistical analyses, with higher scores indicating stronger physical activity motivation. In the present sample, the total composite showed good internal consistency, Cronbach’s *α* = 0.916, and the subscale α values ranged from 0.838 to 0.870. A second-order CFA showed acceptable fit, *χ*^2^/df = 3.586, RMSEA = 0.038, CFI = 0.985, and TLI = 0.982, supporting use of the overall composite score.

#### 3.2.3 Self-regulatory fatigue

Self-regulatory fatigue was measured using a Chinese-language item set based on the Self-Regulatory Fatigue Scale (SRF-18), which assesses individuals’ self-regulatory fatigue during cognitive, emotional, and behavioral regulation (Nes et al., 2013). The scale contains 18 items across three dimensions: Cognitive, Emotional, and Behavioral. Sample items include “I feel full of energy” and “I get easily upset.” The scale used a 5-point agreement response format, ranging from 1 = “strongly disagree” to 5 = “strongly agree.” Items 1, 2, 5, 9, 10, 12, 15, and 18 were reverse scored. After reverse scoring, the mean score of the 18 items was calculated, with higher scores indicating higher levels of self-regulatory fatigue. In the present sample, the total composite showed good internal consistency, Cronbach’s α = 0.921, and the subscale α values ranged from 0.837 to 0.882. A second-order CFA showed good fit, *χ*^2^/df = 2.062, RMSEA = 0.024, CFI = 0.990, and TLI = 0.989, supporting use of the overall composite score.

#### 3.2.4 Aggression

Aggression was measured using a Chinese-language item set derived from the Brief Aggression Questionnaire (BAQ), which assesses individuals’ aggressive tendencies (Webster et al., 2014). The scale contains 12 items across four dimensions: Physical Aggression, Anger, Verbal Aggression, and Hostility, with three items in each dimension. Sample items include “Given enough provocation, I may hit another person” and “Sometimes I fly off the handle for no good reason.” The original BAQ uses a 7-point self-descriptiveness response scale. To standardize the response format across the questionnaire, this study modified the response options to a 5-point self-descriptiveness scale, ranging from 1 = “very unlike me” to 5 = “very like me.” Therefore, the scores in this study were used only as continuous indicators of aggression in the present sample and were not directly compared with scores or norms based on the original 7-point BAQ. Item 4 was reverse scored. After reverse scoring, the mean score of the 12 items was calculated, with higher scores indicating higher levels of aggression. In the present sample, the total composite showed good internal consistency, Cronbach’s *α* = 0.909, and the subscale α values ranged from 0.878 to 0.894. A second-order CFA showed good fit, *χ*^2^/df = 2.791, RMSEA = 0.032, CFI = 0.993, and TLI = 0.991, supporting use of the overall composite score.

### 3.3 Statistical analysis

Data were analyzed using IBM SPSS Statistics 26.0, AMOS 26.0, and the PROCESS macro. No missing values were present in the final analytic sample. First, reverse-scored items were recoded. CFA models were estimated in AMOS 26.0 using maximum likelihood and included the single-factor anxiety model, the second-order physical activity motivation, self-regulatory fatigue, and aggression models, and the hierarchical four-construct model. A comparison model in which all 52 items loaded on one common factor was also estimated; no correlated residuals or post-hoc model modifications were specified. Model fit was evaluated primarily using *χ*^2^/df, CFI, TLI, and RMSEA; CFI and TLI values of at least 0.90 and RMSEA values of no more than 0.08 were considered acceptable. Mean scale scores for anxiety, physical activity motivation, self-regulatory fatigue, and aggression were then calculated and used as composite scores. Composite scores were used in the regression and PROCESS analyses because the purpose of this study was to test prespecified statistical indirect associations at the observed-variable level rather than to construct a full latent-variable structural equation model (SEM). Use of the overall physical activity motivation, self-regulatory fatigue, and aggression composites was additionally supported by their total-score reliability and corresponding second-order CFA models. Cronbach’s *α* was used to assess internal consistency, whereas Harman’s single-factor test and the one-factor CFA comparison were used as preliminary common-method diagnostics; neither was interpreted as ruling out common-method variance. Descriptive analyses included independent-samples *t* tests with Cohen’s *d*, one-way ANOVAs with partial eta squared and Tukey HSD tests, Pearson correlations with Fisher-transformed 95% confidence intervals, and partial correlations controlling for gender and age-group indicators. Gender was coded as 1 = male and 2 = female. Age was represented by indicator variables for age 19, age 20, and age 21 years or older, with age 18 as the reference group. Grade level was not additionally included because it was not prespecified as a covariate and conceptually overlapped with age group as an indicator of educational stage. Regression assumptions and influential observations were examined using residual plots, tolerance and VIF values, Cook’s distance, leverage, and studentized residuals. No separate multiplicity correction was applied to the prespecified egression paths. Statistical indirect associations were estimated using PROCESS Model 6, with anxiety as the independent variable, aggression as the dependent variable, and physical activity motivation and self-regulatory fatigue entered sequentially as prespecified statistical intermediary variables. This model estimated two single-intermediary statistical indirect associations and one prespecified serial statistical indirect association. Gender and the three age-group indicator variables were included as covariates. Statistical indirect associations were estimated using 5,000 percentile bootstrap resamples and were considered statistically distinguishable from zero when their 95% confidence intervals did not include zero. Completely standardized statistical indirect association estimates were also reported. All tests were two-tailed, and the significance level was set at *p* < 0.05. As a competing-order analysis, the intermediary-variable order was reversed while retaining the same model specification, covariates, and bootstrap settings. As an additional sensitivity analysis, the prespecified model was re-estimated after excluding observations with absolute studentized residuals greater than 3; the full-sample analysis remained primary. Given the cross-sectional design, all PROCESS estimates were interpreted as statistical associations and not as evidence of causal mediation, temporal ordering, or confirmed psychological mechanisms.

## 4 Results

### 4.1 Descriptive statistics of anxiety, physical activity motivation, self-regulatory fatigue, and aggression

Table 1 presents the descriptive statistics for anxiety, physical activity motivation, self-regulatory fatigue, and aggression. The overall mean scores for the four variables were 3.36 ± 0.81, 3.25 ± 0.50, 3.23 ± 0.48, and 3.25 ± 0.52, respectively. Statistically significant gender differences were observed in all four variables, although the corresponding Cohen’s *d* values indicated small effects. Specifically, male students scored higher on anxiety and self-regulatory fatigue, whereas female students scored higher on physical activity motivation and aggression. The omnibus one-way ANOVAs indicated statistically significant age-group differences in all four variables, although the corresponding partial eta-squared values were small. Tukey HSD tests indicated that students aged 21 years or older reported higher anxiety than those aged 18 or 19 years, students aged 18 years reported higher physical activity motivation than those aged 21 years or older, and students aged 21 years or older reported higher aggression than those aged 19 years; no adjusted pairwise difference was statistically significant for self-regulatory fatigue. These descriptive findings provided context for the subsequent correlation analyses and tests of statistical indirect associations.

**Table 1:** Descriptive statistics (M ± SD) and group difference tests for anxiety, physical activity motivation, self-regulatory fatigue, and aggression.

Group	*N*	Anxiety	Physical activity motivation	Self-regulatory fatigue	Aggression
Male	855	3.40 ± 0.82	3.21 ± 0.48	3.26 ± 0.49	3.22 ± 0.52
Female	918	3.32 ± 0.80	3.28 ± 0.51	3.20 ± 0.47	3.28 ± 0.52
Overall	1773	3.36 ± 0.81	3.25 ± 0.50	3.23 ± 0.48	3.25 ± 0.52
Gender differences, *t*(1,771)		2.105 (0.035; 0.100)	−2.741 (0.006; −0.130)	2.668 (0.008; 0.127)	−2.501 (0.012; −0.119)
Age differences, *F*(3,1769)		3.933 (0.008; 0.007)	3.015 (0.029; 0.005)	3.097 (0.026; 0.005)	3.068 (0.027; 0.005)

Values are presented as M ± SD. For gender differences, values outside parentheses are signed *t* statistics, and values within parentheses are exact *p* values and Cohen’s *d*, respectively. For age differences, values outside parentheses are *F* statistics, and values within parentheses are exact *p* values and partial eta squared (η_p_^2^), respectively. Cohen’s d was calculated as male minus female; therefore, negative values indicate higher scores among female students. Detailed confidence intervals and age-group post hoc comparisons are reported in the Supplementary material. ANX, anxiety; PAM, physical activity motivation; SRF, self-regulatory fatigue; AGG, aggression; M, mean; SD, standard deviation.

### 4.2 Common-method variance assessment

Because all core variables in this study were obtained from self-report questionnaire data collected at the same time point, Harman’s single-factor test was used as a preliminary assessment of common-method variance. As a preliminary diagnostic, all measurement items were entered simultaneously into a principal component analysis, and the variance explained by the first component in the initial unrotated solution was examined. The results showed that 13 components had eigenvalues greater than 1.0 and that the first unrotated component explained 21.61% of the total variance. This pattern did not indicate that a single unrotated component accounted for most of the item variance. The one-factor CFA comparison model also showed poor fit and was substantially inferior to the hierarchical four-construct model; detailed fit indices are reported in the Supplementary material. Nevertheless, these diagnostics do not rule out common-method variance.

### 4.3 Correlation analysis of anxiety, physical activity motivation, self-regulatory fatigue, and aggression

Table 2 presents the means, standard deviations, and zero-order Pearson correlations with 95% confidence intervals for the study variables. Anxiety was positively correlated with aggression (*r* = 0.260, 95% CI [0.216, 0.303], *p* < 0.001) and self-regulatory fatigue (*r* = 0.254, 95% CI [0.210, 0.297], *p* < 0.001), and negatively correlated with physical activity motivation (*r* = −0.257, 95% CI [−0.300, −0.213], *p* < 0.001). Aggression was negatively correlated with physical activity motivation (*r* = −0.256, 95% CI [−0.299, −0.212], *p* < 0.001) and positively correlated with self-regulatory fatigue (*r* = 0.263, 95% CI [0.219, 0.306], *p* < 0.001). Physical activity motivation was also negatively correlated with self-regulatory fatigue (*r* = −0.238, 95% CI [−0.281, −0.194], *p* < 0.001). Overall, the zero-order correlations were modest in magnitude and were consistent with the hypothesized directions. Partial correlations controlling for gender and the three age-group indicators were materially similar (absolute *r_p_* values = 0.233–0.265, all *p* < 0.001; Supplementary material), providing an adjusted correlational basis for the subsequent statistical indirect-association analyses.

**Table 2:** Means, standard deviations, and zero-order Pearson correlations with 95% confidence intervals among the study variables.

Variable	M	SD	1	2	3	4
Anxiety	3.36	0.81	1			
Physical activity motivation	3.25	0.50	−0.257 [−0.300, −0.213]	1		
Self-regulatory fatigue	3.23	0.48	0.254 [0.210, 0.297]	−0.238 [−0.281,−0.194]	1	
Aggression	3.25	0.52	0.260 [0.216, 0.303]	−0.256 [−0.299,−0.212]	0.263 [0.219, 0.306]	1

*N* = 1,773. Values below the diagonal are zero-order Pearson correlation coefficients, with 95% confidence intervals calculated using Fisher’s z transformation shown in brackets. All tests were two-tailed, and all zero-order correlations were statistically significant at *p* < 0.001. Partial correlations controlling for gender and three age-group indicator variables, with age 18 as the reference category, were materially similar and are reported in the Supplementary material. ANX, anxiety; PAM, physical activity motivation; SRF, self-regulatory fatigue; AGG, aggression; M, mean; SD, standard deviation.

### 4.4 Testing statistical indirect associations involving physical activity motivation and self-regulatory fatigue in the anxiety–aggression association

**Figure 2:**
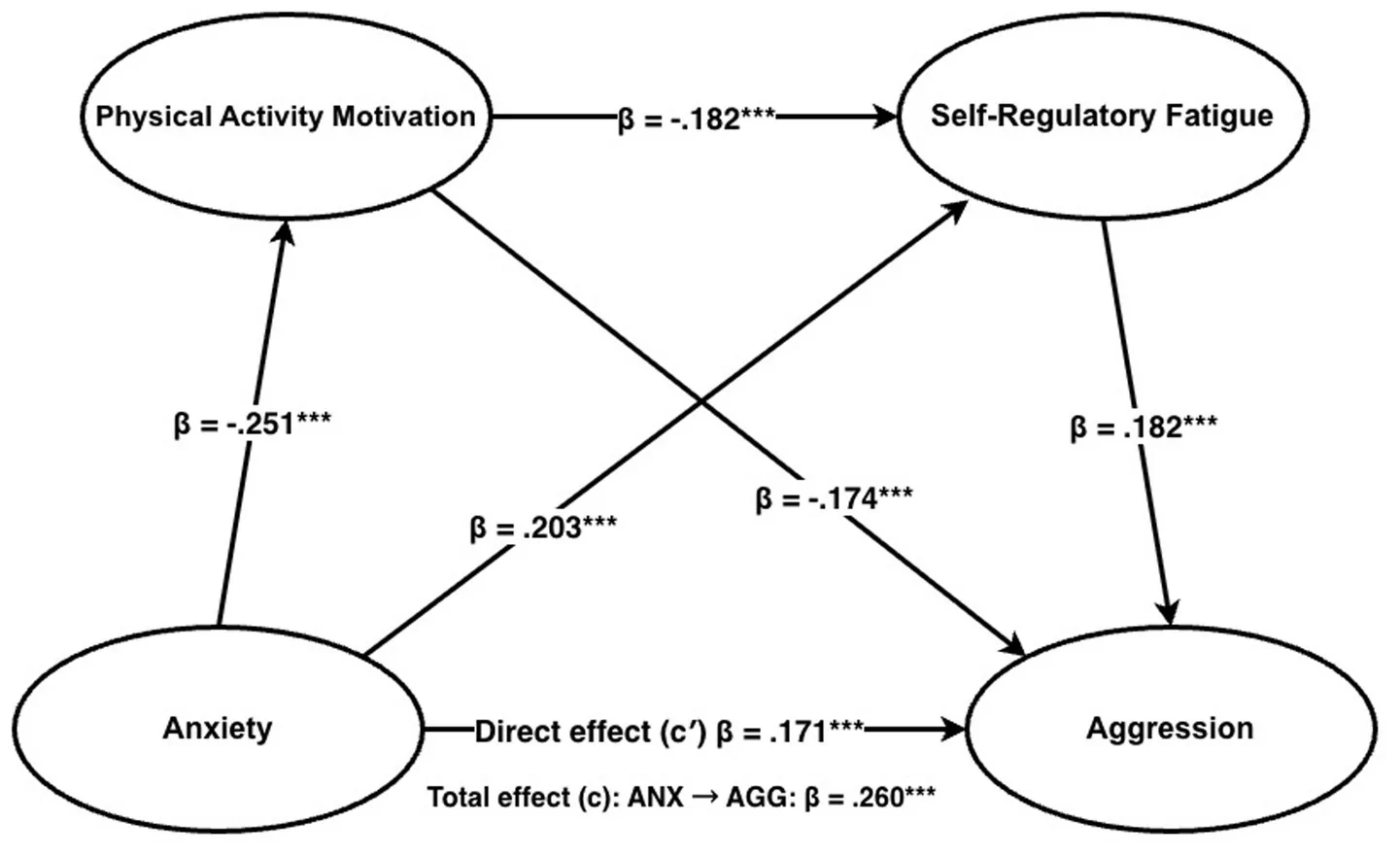
Standardized regression coefficients for the prespecified cross-sectional statistical association model. All variables were observed composite scores. Gender and three age-group indicator variables, with age 18 as the reference category, were included as covariates in all regression equations but are not shown. The direct association (c′) represents the anxiety–aggression association after physical activity motivation and self-regulatory fatigue were included as prespecified statistical intermediary variables, whereas the total association (c) represents the anxiety–aggression association before their inclusion. ANX, anxiety; PAM, physical activity motivation; SRF, self-regulatory fatigue; AGG, aggression. The coefficients represent cross-sectional statistical associations and do not imply causal effects or temporal precedence. ***p < 0.001.

With anxiety specified as the independent variable, aggression as the dependent variable, and physical activity motivation and self-regulatory fatigue as prespecified statistical intermediary variables, PROCESS Model 6 was used to estimate the specific and serial statistical indirect associations within the anxiety–aggression association. Gender and the three age-group indicator variables were included as covariates, with age 18 as the reference group. Before testing these statistical indirect effects, the four-construct measurement model tested in AMOS showed good fit, χ^2^/df = 1.434, RMSEA = 0.016, CFI = 0.989, TLI = 0.988, supporting the distinctiveness of the four constructs: anxiety, physical activity motivation, self-regulatory fatigue, and aggression. The regression results are presented in Table 3, and the standardized regression coefficients are shown in Figure 2. Anxiety was negatively associated with physical activity motivation (*β* = −0.251, *p* < 0.001) and positively associated with self-regulatory fatigue (*β* = 0.203, *p* < 0.001) and aggression (*β* = 0.171, *p* < 0.001). Physical activity motivation was negatively associated with self-regulatory fatigue (*β* = −0.182, *p* < 0.001) and aggression (*β* = −0.174, *p* < 0.001). Self-regulatory fatigue was significantly and positively associated with aggression (*β* = 0.182, *p* < 0.001). When the two statistical intermediary variables were not included, the total association between anxiety and aggression was also statistically significant (*β* = 0.260, *p* < 0.001). All regression equations were statistically significant. The models explained 7.1, 10.1, and 14.5% of the variance in physical activity motivation, self-regulatory fatigue, and aggression, respectively, and the total-association model explained 7.6% of the aggression variance, corresponding to R^2^ = 0.071, 0.101, 0.145, and 0.076. Table 4 presents the bootstrap estimates of the statistical indirect associations, together with the direct and total associations between anxiety and aggression. The total statistical indirect association was *B* = 0.0570, 95% bootstrap CI [0.0452, 0.0696]. The statistical indirect association involving physical activity motivation was *B* = 0.0279, 95% bootstrap CI [0.0196, 0.0371]. The statistical indirect association involving self-regulatory fatigue was *B* = 0.0238, 95% bootstrap CI [0.0163, 0.0321]. The serial statistical indirect association involving physical activity motivation followed by self-regulatory fatigue was *B* = 0.0053, 95% bootstrap CI [0.0034, 0.0077]; its completely standardized estimate was 0.0083, 95% bootstrap CI [0.0053, 0.0120], indicating a small magnitude. After physical activity motivation and self-regulatory fatigue were included, the direct association between anxiety and aggression remained statistically significant, *B* = 0.1101, 95% CI [0.0807, 0.1395], whereas the total association was *B* = 0.1671, 95% CI [0.1382, 0.1961]. Together, the bootstrap confidence intervals for the two single-intermediary and one serial statistical indirect associations excluded zero, while the direct association also remained statistically distinguishable from zero. Thus, the observed results were consistent with the predictions of H2, H3, and H4. In the competing-order analysis, the reversed sequence involving self-regulatory fatigue followed by physical activity motivation also yielded a small serial statistical indirect association, *B* = 0.0051, 95% bootstrap CI [0.0032, 0.0073], with a completely standardized estimate of 0.0079. After four observations with absolute studentized residuals greater than 3 were excluded, all specific statistical indirect associations remained distinguishable from zero, and the serial estimate remained small, *B* = 0.0056, 95% bootstrap CI [0.0035, 0.0081]. Given the cross-sectional design and the compatibility of both intermediary-variable orderings with the data, these results should not be interpreted as evidence of causal relationships or temporal precedence.

**Table 3:** Regression estimates for the prespecified cross-sectional statistical association model.

Variable	Physical activity motivation		Self-regulatory fatigue		Aggression		Total association	
	B (SE) [95% CI]	*β*; *t*; *p*	B (SE) [95% CI]	*β*; *t*; *p*	B (SE) [95% CI]	*β*; *t*; *p*	B (SE) [95% CI]	*β*; *t*; *p*
Anxiety	−0.155 (0.014) [−0.183, −0.127]	−0.251; −10.885; <0.001	0.121 (0.014) [0.094, 0.149]	0.203; 8.682; <0.001	0.110 (0.015) [0.081, 0.139]	0.171; 7.352; <0.001	0.167 (0.015) [0.138, 0.196]	0.260; 11.328; <0.001
Physical activity motivation	—	—	−0.176 (0.023) [−0.221, −0.132]	−0.182; −7.788; <0.001	−0.180 (0.024) [−0.228, −0.133]	−0.174; −7.470; <0.001	—	—
Self-regulatory fatigue	—	—	—	—	0.196 (0.025) [0.147, 0.245]	0.182; 7.845; <0.001	—	—
*R* ^2^	0.071		0.101		0.145		0.076	
Adjusted *R*^2^	0.069		0.098		0.142		0.073	
*F*	*F*(5,1767) = 27.199, (*p* < 0.001)		*F*(6,1766) = 33.203, (*p* < 0.001)		*F*(7,1765) = 42.854, (*p* < 0.001)		F(5,1767) = 28.979, (*p* < 0.001)	

*N* = 1,773. For each model, the first subcolumn presents the unstandardized coefficient, standard error, and 95% confidence interval in the format B (SE) [95% CI]. The second subcolumn presents the standardized coefficient, signed *t* statistic, and *p* value in the format *β*; *t*; *p*. Gender and three age-group indicator variables were included as covariates in all models, with age 18 as the reference category. Gender was coded as 1 = male and 2 = female. The full aggression model included anxiety, physical activity motivation, and self-regulatory fatigue simultaneously, whereas the total-association model estimated the anxiety–aggression association before physical activity motivation and self-regulatory fatigue were included. Full covariate coefficients are reported in the Supplementary material. Given the cross-sectional design, the regression coefficients represent statistical associations and should not be interpreted as causal effects. *B*, unstandardized coefficient; SE, standard error; *β*, standardized coefficient; CI, confidence interval; PAM, physical activity motivation; SRF, self-regulatory fatigue.

**Table 4:** Total and direct associations and bootstrap estimates of statistical indirect associations between anxiety and aggression.

Association pattern	Unstandardized estimate, *B* (SE/BootSE)	95% CI	Completely standardized estimate	Standardized 95% bootstrap CI
ANX → PAM → AGG	0.0279 (0.0045)	[0.0196, 0.0371]	0.0435	[0.0307, 0.0575]
ANX → SRF → AGG	0.0238 (0.0041)	[0.0163, 0.0321]	0.0370	[0.0256, 0.0501]
ANX → PAM → SRF → AGG	0.0053 (0.0011)	[0.0034, 0.0077]	0.0083	[0.0053, 0.0120]
Total statistical indirect association	0.0570 (0.0062)	[0.0452, 0.0696]	0.0888	[0.0712, 0.1080]
Direct association (c′)	0.1101 (0.0150)	[0.0807, 0.1395]	0.1714	—
Total association (c)	0.1671 (0.0148)	[0.1382, 0.1961]	0.2603	—

*N* = 1,773. Unstandardized statistical indirect associations were estimated using percentile bootstrap confidence intervals based on 5,000 bootstrap samples. For the indirect-association rows, the values in parentheses are bootstrap standard errors (BootSE), and both unstandardized and completely standardized confidence intervals are percentile bootstrap intervals. For the total and direct associations, the values in parentheses are model-based standard errors (SE), and the reported confidence intervals are model-based 95% confidence intervals. Gender and three age-group indicator variables were included as covariates, with age 18 as the reference category. The direct association remained statistically significant after physical activity motivation and self-regulatory fatigue were included. The serial completely standardized statistical indirect association was small in magnitude. Given the cross-sectional design, none of the estimates should be interpreted as evidence of causal mediation or temporal ordering. ANX, Anxiety; PAM, Physical Activity Motivation; SRF, Self-Regulatory Fatigue; AGG, aggression; *B*, unstandardized estimate; SE, standard error; BootSE, bootstrap standard error; CI, confidence interval.

## 5 Discussion

This study examined the anxiety–aggression association among students at one medical college in China and tested single-intermediary and serial statistical indirect associations involving physical activity motivation and self-regulatory fatigue. Overall, the zero-order correlations were modest, and the final aggression model explained 14.5% of the variance. The two single-intermediary statistical indirect associations were larger than the prespecified serial association, whose completely standardized estimate was 0.0083, indicating a small magnitude. The competing-order analysis yielded a similarly small serial association, indicating that the cross-sectional data did not distinguish whether physical activity motivation or self-regulatory fatigue came first. Given the large sample and cross-sectional design, statistical significance should be interpreted together with effect magnitude, and none of the estimates establishes causal or temporal processes.

### 5.1 Anxiety and aggression

Anxiety showed a modest positive association with aggression, and the adjusted direct association remained statistically distinguishable from zero, consistent with H1. This result is broadly consistent with previous research. Related studies have shown that higher anxiety is often associated with aggression-related components such as generalized aggression, anger, and hostility, suggesting that anxiety-related experiences may be accompanied not only by avoidance or withdrawal but also by irritability, hostility, and aggressive tendencies (Florek et al., 2023). A similar pattern has been reported in clinical samples, although such evidence is contextual rather than directly comparable with the present medical-college sample (Park and Seo, 2018). Among college students, different combinations of social anxiety and impulsivity have also been shown to distinguish levels of aggression, with students characterized by both high social anxiety and high impulsivity tending to show higher aggressive tendencies (Zhang et al., 2024). Within the General Aggression Model, heightened arousal, threat sensitivity, hostile interpretation, and impulsive responding provide possible interpretations of the observed association, but these processes were not measured directly.

The association between anxiety and aggression may not reflect a single psychological process, but may involve the joint roles of social cognition, interpersonal stress, and contextual experiences. The observed association may also partly reflect unmeasured common causes, including depression, impulsivity, academic stress, sleep problems, hostile attribution, and peer victimization. Previous research has suggested that threat interpretations related to social anxiety and paranoia can jointly predict aggressive behavior, indicating that individuals with higher anxiety may be more likely to interpret ambiguous interpersonal cues as threatening and may therefore show defensive or aggressive responses (Mallott et al., 2024). Evidence from Chinese adolescent samples further suggests that social anxiety may be linked to later aggression, although this evidence comes from a younger population and does not establish the same pattern among college students (Zhang et al., 2023). Longitudinal research has further shown dynamic associations among social anxiety, peer victimization, and aggression at both trait and state levels, suggesting that anxiety and aggression may be dynamically linked in interpersonal interaction processes (Zhou et al., 2024). In this medical-college sample, academic evaluation, interpersonal expectations, dormitory relationships, and uncertainty about future development are plausible contextual influences; however, these contextual and cultural processes were not measured. Because this study used a cross-sectional self-report questionnaire design, these findings should be interpreted as statistical associations rather than evidence of causality. Future research could employ longitudinal designs, cross-lagged panel models, or experience sampling methods to examine the temporal ordering, bidirectional associations, and contextual variation between anxiety and aggression. Future studies could include depression, academic stress, sleep quality, hostile attribution, threat interpretation, impulsivity, emotion regulation, and peer victimization to provide a more complete account of the anxiety–aggression association.

### 5.2 Independent statistical indirect association involving physical activity motivation

The statistical indirect association involving physical activity motivation (PAM) was distinguishable from zero and was consistent with H2. This pattern is compatible with PAM as one correlate of the anxiety–aggression association rather than as an established psychological mechanism. From the perspective of Self-Determination Theory, one possible interpretation is that anxiety-related threat and evaluation concerns are associated with less favorable autonomy, competence, and relatedness experiences in physical-activity contexts. Previous research has also shown that, under stressful or restrictive conditions, basic psychological need satisfaction, self-determined motivation, affect, and anxiety are closely related, indicating that anxiety states may be associated with changes in physical activity motivational quality and intention (Jang et al., 2021; Antunes et al., 2024). Furthermore, PAM not only reflects individuals’ tendency to engage in physical activity but may also represent a positive psychological resource. Among college students, physical activity motivation has been associated with multiple dimensions of psychological well-being, suggesting that higher PAM may be linked to better psychological adjustment, a stronger sense of purpose in life, and more positive self-experiences (Zhong, 2024). In the present study, lower PAM may mark fewer positive physical-activity experiences or other correlated resources, but the data do not show that anxiety reduced PAM or that lower PAM caused aggression.

The association between PAM and lower aggression may also be related to positive psychological resources such as self-efficacy, mental toughness, leisure satisfaction, and self-control. Because self-efficacy, mental toughness, leisure satisfaction, self-control, and actual physical activity were not measured, they also remain alternative explanations for the PAM–aggression association. Previous research among college students has shown that exercise motivation may be linked to self-efficacy through leisure satisfaction and mental toughness, suggesting that higher exercise motivation may be accompanied by stronger self-efficacy, mental toughness, and stress-coping resources (Yu et al., 2024). Physical activity research also provides bridging evidence for this interpretation. For example, physical activity level may be associated with lower aggressive behavior through self-efficacy and self-control, and physical activity interventions have been linked to reductions in aggressive behaviors (Yu et al., 2024; Ouyang and Liu, 2023). Taken together, these studies provide indirect contextual support, but they do not establish a direct PAM → aggression process in this sample. Importantly, this study measured PAM rather than actual physical activity level or intervention effects. Thus, the present findings should be interpreted as the statistical indirect effect of PAM and not as evidence that physical activity behavior causally affects aggression. Future research could include PAM, actual physical activity, exercise adherence, and physical activity experiences to distinguish the relative roles of “motivation” and “behavior” in the anxiety–aggression association.

### 5.3 Independent statistical indirect association involving self-regulatory fatigue

The statistical indirect association involving self-regulatory fatigue (SRF) was distinguishable from zero and was consistent with H3. This pattern is compatible with SRF as one correlate of the anxiety–aggression association rather than as an established mechanism. From the perspective of the Strength Model of Self-Control, anxiety is often accompanied by persistent worry, emotional arousal, attentional control demands, and efforts to suppress rumination. These effortful regulatory demands could be associated with greater cognitive, emotional, and behavioral fatigue, although this temporal process was not tested. Previous research has also shown that academic anxiety is closely associated with SRF among college students, and that academic stress is related to higher levels of SRF in nursing student samples (Kong et al., 2025; Zhang et al., 2022). In addition, research on threat coping has suggested that coping with threat may produce self-control spillover and be related to subsequent behavioral outcomes such as aggression, attention, and decision making (Inzlicht and Kang, 2010). In this medical-college sample, academic demands, interpersonal evaluation, and persistent worry may be relevant contextual correlates, but the data do not show that anxiety temporally preceded SRF.

The association between SRF and higher aggression can be understood in relation to the inhibition component proposed in I^3^ Theory. According to this theory, whether aggressive responses are expressed depends not only on instigation and impellance, but also on whether individuals have sufficient inhibitory capacity. Higher SRF may theoretically correspond to weaker inhibition or response delay during conflict, but inhibitory capacity was not measured directly. Depressive symptoms, sleep quality, academic stress, and impulsivity may also contribute to both SRF and aggression. Previous research on ego depletion and aggressive behavior, as well as a meta-analysis of self-control and aggression among Chinese students, provides bridging evidence for this interpretation, suggesting that insufficient regulatory resources or lower self-control may be associated with higher aggressive tendencies (Barlett et al., 2016; Lei et al., 2020). Experience sampling research has further shown dynamic associations between self-regulatory control processes and aggressive ideations and behaviors in daily contexts (Plessen et al., 2023). Importantly, this study measured SRF rather than general self-control or laboratory-induced ego depletion. Thus, the present findings should be interpreted as the statistical indirect effect of SRF and not as evidence that regulatory fatigue causally affects aggression. Future research could combine longitudinal designs, behavioral inhibition tasks, experience sampling methods, or physiological indicators to examine the temporal ordering, contextual variation, and dynamic processes of SRF in the anxiety–aggression association.

### 5.4 Serial statistical indirect association involving physical activity motivation and self-regulatory fatigue

The prespecified serial statistical indirect association involving PAM followed by SRF was distinguishable from zero and was consistent with H4, but its completely standardized estimate (0.0083) was small. The competing-order model produced a similarly small serial association, so the data did not identify PAM → SRF as a unique or temporally prior ordering. One theory-compatible interpretation is that anxiety-related experiences are associated with less favorable motivational experiences in physical-activity contexts. Previous research has also shown close associations among anxiety, motives, and intention for physical activity (Galli et al., 2022). Further, from the perspective of Conservation of Resources Theory, PAM can be understood as a physical activity–related motivational psychological resource. Higher PAM may indicate that students are more likely to obtain psychological resource support through exercise goals, positive experiences, perceived competence, and self-regulatory engagement. In contrast, lower PAM may be associated with fewer opportunities for resource recovery and greater regulatory load. Related studies have shown that autonomous motivation and autonomous fitness behavior are associated with self-regulatory resources such as effortful self-control and exercise identity (Bourque et al., 2025; Chen and Wang, 2025), and aerobic exercise may also provide behavioral-level bridging evidence for self-control recovery after ego depletion (Zou et al., 2016). These studies provide contextual support for an association between PAM and SRF, but they do not establish temporal precedence. However, this study measured physical activity motivation rather than actual physical activity level or exercise intervention effects.

In the latter part of the pathway, the connection between SRF and aggression can be interpreted in relation to the inhibition component proposed in I^3^ Theory. Higher SRF may theoretically be related to weaker inhibition during frustration or conflict, but this process was not observed directly. Previous research has shown that angry rumination and reduced self-control capacity are important processes in the provocation–aggression association, and that response inhibition deficits are associated with reactive aggression (Denson et al., 2011; Sun et al., 2020). At the same time, a related serial model showed that sport motivation was linked to lower aggression through emotional intelligence and self-control, providing proximal evidence for the explanatory direction of “sport motivation–regulatory resources–aggression” (Wang et al., 2026). However, that study did not include anxiety or SRF; therefore, it should be regarded as support for a related explanatory pattern rather than a direct replication of the present model. Because this study was cross-sectional and both intermediary-variable orderings were compatible with the data, the serial results should be interpreted as alternative statistical descriptions rather than evidence of a causal sequence. The small standardized estimate also indicates that statistical significance, aided by the large sample, should not be equated with practical importance. Whether the serial association differs by gender, age, or other student characteristics remains unknown. Overall, the observed covariance pattern is hypothesis-generating and supports further longitudinal testing of motivation–regulation models rather than a confirmed sequence from anxiety to aggression.

## 6 Practical implications

This study provides preliminary, hypothesis-generating implications for mental health education, physical activity–based health promotion, and future research on aggression-related risk in higher education settings. The findings suggest that, in this medical-college sample, the anxiety–aggression association co-occurred with lower physical activity motivation and higher self-regulatory fatigue. Here, a cross-domain perspective refers specifically to considering anxiety-related experiences, physical activity motivation, and self-regulatory fatigue within the same statistical framework. At the applied level, these variables may be considered together when developing and prospectively evaluating coordinated approaches involving mental health education, physical education, and student services. The present study did not evaluate screening accuracy, intervention effectiveness, actual physical activity, or aggression prevention; therefore, these applications should not be treated as validated intervention recommendations.

For future program development, the PAM findings suggest examining not only whether students participate in physical activity, but also why they participate, how they maintain participation, and whether they obtain positive experiences from physical activity. Candidate approaches for prospective evaluation include diverse, low-pressure, and optional forms of physical activity, such as aerobic, mind–body, outdoor, and team-based activities, tailored to students’ interests, abilities, and social needs. Physical education courses could also reduce reliance on physical fitness test scores, ranking comparisons, or external pressure, and instead support students’ autonomy and competence through autonomous choice, graded goals, positive feedback, and skill support. In addition, group cooperation, peer support, and noncompetitive physical activity contexts may help strengthen students’ relatedness and make physical activity a context for emotional support and belonging. For students with higher anxiety, lower willingness to exercise, or poorer physical activity experiences, gradual, encouraging, and interest-oriented participation approaches could be evaluated for their potential to reduce participation pressure and support more stable physical activity motivation. Because this study measured motivation rather than actual physical activity or intervention outcomes, these suggestions concern environments to be evaluated and do not constitute exercise prescriptions or evidence of effectiveness.

The statistical indirect association involving self-regulatory fatigue suggests that SRF may warrant examination in future research on anxiety-related aggression risk, but the present study did not establish screening validity. These contextual exposures and behavioral manifestations were not measured directly in the present study. Students chronically exposed to academic pressure, interpersonal evaluation, dormitory conflict, or uncertainty about the future may experience greater cognitive, emotional, and behavioral regulatory load, which may be reflected in irritability, difficulty with impulse control, or increased interpersonal conflict risk. Future intervention studies could evaluate whether support delivered through counseling, physical education, and student services—such as emotion-regulation, stress-recovery, mindfulness, sleep and time-management, impulse-control, and physical-activity-motivation components—improves relevant outcomes. Overall, the findings motivate a hypothesis-generating framework for future research that integrates attention to anxiety-related experiences, support for physical activity motivation, consideration of self-regulatory fatigue, and aggression-related outcomes. The usefulness and sequencing of this framework should be tested prospectively in longitudinal or intervention studies before implementation claims are made.

## 7 Limitations and future directions

Several limitations should be acknowledged. These limitations concern measurement methods, study design, potential confounding, model robustness, sample characteristics, and the generalizability of the findings. These limitations should be considered alongside the study’s large sample, prespecified hypotheses, bootstrap estimation, and additional robustness analyses.

### 7.1 Limitations of measurement methods

The measurements in this study relied primarily on self-report questionnaires collected at a single time point, which may have introduced social desirability bias, self-perception bias, and common method bias. Harman’s single-factor test and the one-factor CFA comparison were only preliminary diagnostics and cannot rule out common-method variance. Future research could use marker variables, latent method factor approaches, temporal separation, or multi-source data to more rigorously assess and reduce common method bias. In addition, the GAD-7-derived item set omitted the original two-week recall period and changed the 0–3 symptom-frequency response format to a five-point agreement format, while the BAQ response format was changed from seven to five points. Therefore, the resulting scores should not be directly compared with the original cut-off scores, norms, or findings obtained using the original response formats. Although internal consistency and CFA supported structural coherence and use of the composite scores in this sample, the adapted item sets did not undergo a separate formal forward–back translation or independent pilot validation, and equivalence with the original measures or criterion validity was not established. Measurement error in the observed composite scores may also have attenuated or otherwise influenced the estimated associations. Moreover, although gender and age-group differences were reported descriptively, measurement invariance across gender and age groups was not tested; therefore, these group difference results should be interpreted cautiously. Future studies should retain validated response formats where possible and conduct formal translation and cultural-adaptation procedures, criterion validation, measurement-equivalence testing, and measurement-invariance analyses, supplemented by multi-informant, behavioral, or activity-tracking measures where appropriate.

### 7.2 Limitations in study design and causal inference

This study used a cross-sectional questionnaire design and therefore can only reveal statistical associations among anxiety, physical activity motivation, self-regulatory fatigue, and aggression. It cannot establish temporal ordering or causal direction among the variables. PROCESS Model 6 was used only to estimate prespecified cross-sectional statistical indirect associations, and the competing-order analysis showed that both intermediary-variable sequences were compatible with the data. For example, aggression-related conflict could contribute to later anxiety, and higher self-regulatory fatigue could reduce willingness for physical activity just as lower physical activity motivation could be associated with greater fatigue. Future research could use longitudinal designs, cross-lagged panel models, experience sampling methods, or intervention studies to further examine dynamic associations, temporal ordering, and potential causal directions among these variables.

### 7.3 Limited adjustment for confounding factors

This study controlled for gender and three age-group indicator variables in the statistical models. Given the limited covariate adjustment, the observed regression paths should not be interpreted as independent psychological mechanisms. However, several potential confounding factors were not included, such as depression, impulsivity, hostile attribution, emotion regulation, peer victimization, family functioning, social support, academic stress, sleep quality, grade level, and actual physical activity level. These factors may simultaneously affect anxiety, physical activity motivation, self-regulatory fatigue, and aggression, thereby limiting the explanatory sufficiency of the model. Future research should prespecify a broader covariate framework, directly measure theoretically relevant confounders, and use appropriate sensitivity or latent-variable analyses to evaluate the robustness of the associations.

### 7.4 Model robustness and hypothesis testing

This study used AMOS to test the measurement models and PROCESS Model 6 to examine single-intermediary and serial statistical indirect associations. However, model robustness could be further improved. First, the subsequent path analyses were based on scale composite scores rather than latent variable structural equation modeling (SEM), and therefore measurement error and structural paths were not estimated simultaneously. Second, the revised analyses compared the hierarchical four-construct model with a one-factor CFA model and examined a competing intermediary-variable ordering. An outlier sensitivity analysis also produced similar substantive conclusions, but cross-group stability and measurement invariance were not tested. In particular, the prespecified serial statistical indirect association was very small in magnitude (completely standardized estimate = 0.0083), and its statistical significance may partly reflect the large sample. Age was collected in broad categories, including an open-ended category of 21 years or older; indicator coding avoided treating these categories as a linear continuous measure but could not recover exact age differences. Future research could use latent-variable SEM, exact-age measurement, preregistered competing models, and multigroup or invariance analyses to improve the robustness and reproducibility of model interpretation.

### 7.5 Sample and generalizability

The sample was drawn from Shangqiu Medical College in Henan Province, China, using convenience sampling combined with classroom-based group recruitment. Because the sample source was relatively concentrated, the findings should not be directly generalized to other regions, types of universities, or non-college-student populations. Students at a medical college may differ from non-medical students in academic demands, training context, stress exposure, and demographic composition. In addition, because the survey was administered in classroom settings, observations may have been non-independent within classes or courses. However, this study did not retain class- or course-level identifiers, preventing the use of cluster-robust standard errors or multilevel models. If responses were correlated within classes or courses, conventional standard errors may have been underestimated, potentially overstating statistical precision. At the same time, no *a priori* sample size estimation was conducted before data collection; the sample size was mainly determined by survey accessibility and questionnaire response. Future research should use multi-region and multi-institution samples, stratified or probability-based recruitment where feasible, and designs that retain class-level identifiers for cluster-robust or multilevel analyses.

### 7.6 Future research directions and applications

Future research could extend this work in several directions. At the measurement level, future studies could simultaneously include physical activity motivation, actual physical activity level, exercise adherence, wearable-device-based activity data, and peer or teacher reports to distinguish the roles of physical activity motivation and actual physical activity behavior. At the research design level, longitudinal designs, cross-lagged panel models, and experience sampling methods could be used to examine dynamic changes and bidirectional associations among anxiety, physical activity motivation, self-regulatory fatigue, and aggression in daily contexts. At the model-extension level, future studies could incorporate additional psychological, interpersonal, and behavioral variables, preregister competing models, and examine whether the associations are stable across student subgroups. At the practical-application level, integrated psychological and physical activity–based programs could be prospectively tested to determine whether addressing physical activity motivation and self-regulatory fatigue changes anxiety- and aggression-related outcomes before implementation or prevention claims are made.

## 8 Conclusion

This study examined the anxiety–aggression association among students at one medical college in China, together with single-intermediary and serial statistical indirect associations involving physical activity motivation and self-regulatory fatigue. Anxiety showed a modest positive association with aggression, and the bootstrap results identified statistical indirect associations involving lower physical activity motivation and higher self-regulatory fatigue. The final aggression model explained 14.5% of the variance, indicating modest explanatory power. The observed covariance pattern was compatible with the prespecified ordering, but the serial association was small in magnitude (completely standardized estimate = 0.0083), and a competing reverse ordering yielded a similarly small association.

These findings are compatible with a motivation–regulation perspective in which anxiety-related experiences, physical activity motivation, self-regulatory fatigue, and aggression covary. Drawing on Self-Determination Theory, Conservation of Resources Theory, and self-regulation-related perspectives, the proposed framework should be regarded as hypothesis-generating rather than as a confirmed explanatory mechanism. The findings also offer preliminary implications for future mental health education, physical education, and student-support program development and evaluation, rather than validated prevention recommendations.

Because the study used cross-sectional self-report data and both intermediary-variable orderings were compatible with the data, the findings cannot establish temporal precedence, dynamic change, or causal relationships. Future research should use longitudinal, cross-lagged, experience-sampling, or intervention designs, together with objective physical activity measures, multi-informant assessments, and multi-region, multi-institution samples, to evaluate temporal ordering, robustness, generalizability, and applied relevance.

## Data Availability

The original contributions presented in the study are included in the article/Supplementary material, further inquiries can be directed to the corresponding author.
