# Supplementary material for: Association between anxiety and aggression among students at a medical college in China: a cross-sectional analysis of indirect associations involving physical activity motivation and self-regulatory fatigue
Source: Front Psychol. 2026 Aug 20;17:1916035. doi: 10.3389/fpsyg.2026.1916035 (PMC13537957; doi:10.3389/fpsyg.2026.1916035)
Supplement: Supplementary file 1 [file Data_Sheet_1.PDF]

Supplementary Material

1 Supplementary Tables

Table S1 Scoring characteristics and internal consistency of the study measures

Measure	Items	Dimensions	Response format used	Reverse-scored items	Composite scoring	Total-score α	Subscale α range
GAD-7-derived anxiety item set	7	1	1–5 agreement	None	Item mean	.892	—
Physical activity motivation	15	5	1–5 agreement	None	Item mean	.916	.838–.870
Self-regulatory fatigue	18	3	1–5 agreement	1, 2, 5, 9, 10, 12, 15, 18	Item mean after reverse scoring	.921	.837–.882
BAQ-derived aggression item set	12	4	1–5 self-descriptiveness	4	Item mean after reverse scoring	.909	.878–.894

Note. *Higher composite scores indicate higher levels of the corresponding construct. The GAD-7-derived item set did not retain the original two-week recall period and used a five-point agreement format rather than the original 0–3 symptom-frequency format. The BAQ-derived item set used five rather than seven response categories. α = Cronbach's alpha.*

Table S2 Confirmatory factor analysis model specifications and fit indices

Panel A. *Model specifications and fit indices*

Model	Specification	χ^2	df	χ^2/df	RMR	RMSEA [90% CI]	CFI	TLI
Anxiety model	Seven anxiety items loading on one factor	40.565	14	2.898	.014	.033 [.021, .045]	.995	.993
Physical activity motivation model	Fifteen items loading on five first-order factors, which loaded on one second-order PAM factor	304.842	85	3.586	.009	.038 [.034, .043]	.985	.982
Self-regulatory fatigue model	Eighteen items loading on three first-order factors, which loaded on one second-order SRF factor	272.218	132	2.062	.009	.024 [.020, .029]	.990	.989
Aggression model	Twelve items loading on four first-order factors, which loaded on one second-order aggression factor	139.563	50	2.791	.007	.032 [.026, .038]	.993	.991
Hierarchical four-construct model	One anxiety factor and second-order PAM, SRF, and aggression factors represented within the four-construct model	1801.167	1256	1.434	.012	.016 [.014, .017]	.989	.988
One-factor comparison model	All 52 items loading on one common factor	34614.432	1274	27.170	.089	.122 [.120, .123]	.324	.297

Panel B. *Standardized loading ranges*

Model	Item-loading range	Higher-order loading range
Anxiety model	.705–.763	—
Physical activity motivation model	.787–.838	.749–.767
Self-regulatory fatigue model	.682–.743	.807–.820
Aggression model	.830–.866	.728–.758
Hierarchical four-construct model	.681–.867	.730–.816
One-factor comparison model	—	—

Note. *N = 1,773. All models were estimated in AMOS 26.0 using maximum likelihood estimation. Reverse-scored items were entered after recoding, as described in Supplementary Table S1. No correlated residuals or post-hoc modification-index adjustments were specified. AMOS reported the root mean square residual (RMR) rather than the standardized root mean square residual (SRMR); RMR values are therefore presented without being relabelled as SRMR. In Panel B, item-loading ranges refer to observed items loading on their corresponding first-order factors, whereas higher-order loading ranges refer to first-order factors loading on the higher-order factor. The one-factor comparison model was used solely as a diagnostic comparison, and its standardized loading range was not summarized. PAM = physical activity motivation; SRF = self-regulatory fatigue; RMSEA = root mean square error of approximation; CI = confidence interval; CFI = comparative fit index; TLI = Tucker–Lewis index.*

Table S3 Detailed gender and age-group comparisons for the principal study variables

Panel A. Gender differences

Variable	Male M ± SD	Female M ± SD	Mean difference [95% CI]	t(df)	p	Cohen's d [95% CI]
Anxiety	3.40 ± .82	3.32 ± .80	.0807 [.0055, .1559]	2.105 (1771)	.035	.100 [.007, .193]
Physical activity motivation	3.2 ± .48	3.28 ± .51	−.0649 [−.1114, −.0185]	−2.741 (1771)	.006	−.130 [−.224, −.037]
Self-regulatory fatigue	3.26 ± .49	3.20 ± .47	.0611 [.0162, .1060]	2.668 (1771)	.008	.127 [.034, .220]
Aggression	3.21 ± .52	3.28 ± .52	−.0616 [−.1098, −.0133]	−2.501 (1771)	.012	−.119 [−.212, −.026]

Panel B. Age-group differences

Variable	Age 18 M [95% CI]	Age 19 M [95% CI]	Age 20 M [95% CI]	Age ≥21 M [95% CI]	F(3,1769)	p	ηp²	Significant Tukey HSD comparisons
Anxiety	3.2797 [3.1878, 3.3716]	3.3342 [3.2658, 3.4025]	3.3412 [3.2741, 3.4084]	3.4764 [3.3957, 3.5571]	3.933	.008	.007	≥21 > 18: Δ = .1967 [.0351, .3583], p = .010; ≥21 > 19: Δ = .1423 [.0053, .2792], p = .038
Physical activity motivation	3.3050 [3.2478, 3.3623]	3.2456 [3.2045, 3.2867]	3.2586 [3.2165, 3.3006]	3.1911 [3.1402, 3.2419]	3.015	.029	.005	18 > ≥21: Δ = .1140 [.0140, .2140], p = .018
Self-regulatory fatigue	3.1887 [3.1306, 3.2468]	3.1916 [3.1512, 3.2320]	3.2536 [3.2145, 3.2927]	3.2680 [3.2194, 3.3167]	3.097	.026	.005	None
Aggression	3.2383 [3.1759, 3.3007]	3.2123 [3.1702, 3.2544]	3.2406 [3.1973, 3.2840]	3.3142 [3.2616, 3.3668]	3.068	.027	.005	≥21 > 19: Δ = .1019 [.0139, .1900], p = .016

Note. *Gender was coded as 1 = male and 2 = female. Mean differences and Cohen's d values in Panel A were calculated as male minus female; negative values therefore indicate higher scores among female students. Age groups were coded as 1 = 18 years, 2 = 19 years, 3 = 20 years, and 4 = 21 years or older. Pairwise comparisons were adjusted using Tukey HSD. Δ = mean difference; ηp² = partial eta squared; CI = confidence interval.*

Table S4 Partial correlations among the principal study variables controlling for gender and age-group indicators

Variable	1	2	3	4
1. Anxiety	1			
2. Physical activity motivation	−.251***	1		
3. Self-regulatory fatigue	.249***	−.233***	1	
4. Aggression	.260***	−.259***	.265***	1

Note. *N = 1,773. Partial correlations controlled for gender and three age-group indicator variables, with age 18 as the reference group. Degrees of freedom = 1,767. All tests were two-tailed. ***p < .001.*

Table S5 Full regression results for the prespecified cross-sectional association model

Panel A. Outcome: physical activity motivation

Predictor	B	SE	β	t	p	95% CI for B
Constant	3.733	.067	—	56.117	< .001	[3.603, 3.864]
Anxiety	−.155	.014	−.251	−10.885	< .001	[−.183, −.127]
Gender	.052	.023	.052	2.255	.024	[.007, .097]
Age 19 vs. age 18	−.049	.035	−.046	−1.397	.163	[−.118, .020]
Age 20 vs. age 18	−.034	.035	−.032	−.976	.329	[−.104, .035]
Age ≥21 vs. age 18	−.082	.038	−.068	−2.173	.030	[−.156, −.008]

R^2 = .071; adjusted R^2 = .069; $F(5, 1767)$ = 27.199, p < .001.

Panel B. Outcome: self-regulatory fatigue

Predictor	B	SE	β	t	p	95% CI for B
Constant	3.433	.105	—	32.546	< .001	[3.227, 3.640]
Anxiety	.121	.014	.203	8.682	< .001	[.094, .149]
Physical activity motivation	−.176	.023	−.182	−7.788	< .001	[−.221, −.132]
Gender	−.039	.022	−.041	−1.792	.073	[−.082, .004]
Age 19 vs. age 18	−.016	.033	−.015	−.468	.640	[−.081, .050]
Age 20 vs. age 18	.047	.034	.045	1.413	.158	[−.018, .113]
Age ≥21 vs. age 18	.034	.036	.029	.953	.341	[−.036, .105]

R^2 = .101; adjusted R^2 = .098; $F(6, 1766)$ = 33.203, p < .001.

Panel C. Outcome: aggression, full model

Predictor	B	SE	β	t	p	95% CI for B
Constant	2.704	.140	—	19.321	< .001	[2.429, 2.978]
Anxiety	.110	.015	.171	7.352	< .001	[.081, .139]
Physical activity motivation	−.180	.024	−.174	−7.470	< .001	[−.228, −.133]
Self-regulatory fatigue	.196	.025	.182	7.845	< .001	[.147, .245]
Gender	.093	.023	.090	4.075	< .001	[.048, .138]
Age 19 vs. age 18	−.040	.035	−.036	−1.136	.256	[−.108, .029]
Age 20 vs. age 18	−.021	.035	−.019	−.598	.550	[−.090, .048]
Age ≥21 vs. age 18	.021	.038	.017	.558	.577	[−.053, .095]

R^2 = .145; adjusted R^2 = .142; $F(7, 1765)$ = 42.854, p < .001.

Panel D. Outcome: aggression, total-association model

Predictor	B	SE	β	t	p	95% CI for B
Constant	2.574	.069	—	37.345	< .001	[2.439, 2.710]
Anxiety	.167	.015	.260	11.328	< .001	[.138, .196]
Gender	.075	.024	.072	3.141	.002	[.028, .121]
Age 19 vs. age 18	−.032	.036	−.029	−.888	.374	[−.104, .039]
Age 20 vs. age 18	−.004	.037	−.004	−.119	.905	[−.076, .067]
Age ≥21 vs. age 18	.045	.039	.036	1.159	.246	[−.031, .122]

R^2 = .076; adjusted R^2 = .073; $F(5, 1767)$ = 28.979, p < .001.

Note. *N = 1,773. Gender was coded as 1 = male and 2 = female. Age 18 was the reference category for the three age-group indicator variables. B = unstandardized coefficient; SE = standard error; β = standardized coefficient; CI = confidence interval.*

Table S6 Summary of regression diagnostics and influential-observation statistics

Outcome/model	Studentized residual range	Maximum Cook's distance	Maximum leverage	Tolerance range	VIF range
Physical activity motivation model	−3.412 to 2.839	.011	.007	.493–.997	1.003–2.028
Self-regulatory fatigue model	−3.467 to 3.070	.008	.012	.492–.994	1.006–2.031
Aggression full model	−3.791 to 3.430	.012	.012	.492–.992	1.008–2.031
Aggression total-association model	−3.344 to 3.142	.009	.007	.493–.997	1.003–2.028

Note. *Residual histograms, normal P–P plots, and standardized residual-versus-predicted plots were inspected for each model. Visual inspection did not indicate marked departures from approximate normality, linearity, or homoscedasticity. No important multicollinearity or highly influential observation was indicated. Four observations in the fully adjusted aggression model had absolute studentized residuals greater than 3; the corresponding sensitivity analysis is reported in Table S7.*

Table S7 Competing-order and outlier sensitivity analyses of statistical indirect associations

Panel A. Prespecified order: ANX → PAM → SRF → AGG

Analysis and path	N	B	BootSE	95% bootstrap CI	Completely standardized estimate	Standardized 95% bootstrap CI
Total indirect	1773	.0570	.0062	[.0452, .0696]	.0888	[.0712, .1080]
ANX → PAM → AGG	1773	.0279	.0045	[.0196, .0371]	.0435	[.0307, .0575]
ANX → SRF → AGG	1773	.0238	.0041	[.0163, .0321]	.0370	[.0256, .0501]
ANX → PAM → SRF → AGG	1773	.0053	.0011	[.0034, .0077]	.0083	[.0053, .0120]

Panel B. Competing order: ANX → SRF → PAM → AGG

Analysis and path	N	B	BootSE	95% bootstrap CI	Completely standardized estimate	Standardized 95% bootstrap CI
Total indirect	1773	.0570	.0061	[.0455, .0696]	.0888	[.0712, .1078]
ANX → SRF → AGG	1773	.0291	.0047	[.0202, .0386]	.0453	[.0315, .0598]
ANX → PAM → AGG	1773	.0229	.0039	[.0157, .0308]	.0356	[.0244, .0481]
ANX → SRF → PAM → AGG	1773	.0051	.0010	[.0032, .0073]	.0079	[.0051, .0114]

Panel C. Outlier sensitivity analysis: prespecified order after excluding four observations

Analysis and path	N	B	BootSE	95% bootstrap CI	Completely standardized estimate	Standardized 95% bootstrap CI
Total indirect	1769	.0584	.0064	[.0463, .0716]	.0917	[.0733, .1110]
ANX → PAM → AGG	1769	.0281	.0045	[.0200, .0372]	.0442	[.0315, .0584]
ANX → SRF → AGG	1769	.0247	.0042	[.0168, .0334]	.0388	[.0266, .0523]
ANX → PAM → SRF → AGG	1769	.0056	.0012	[.0035, .0081]	.0088	[.0056, .0126]

Note. *Statistical indirect associations were estimated using 5,000 percentile bootstrap samples. The outlier sensitivity analysis excluded four observations with absolute studentized residuals greater than 3 in the fully adjusted aggression regression. Gender and three age-group indicator variables were included as covariates in all models. ANX = anxiety; PAM = physical activity motivation; SRF = self-regulatory fatigue; AGG = aggression; BootSE = bootstrap standard error; CI = confidence interval.*
